# T130. ASSOCIATIONS BETWEEN DIFFERENT TYPES OF CHILDHOOD ADVERSITY AND 5-YEAR OUTCOMES IN FIRST-EPISODE PSYCHOSIS PATIENTS

**DOI:** 10.1093/schbul/sby016.406

**Published:** 2018-04-01

**Authors:** Olesya Ajnakina, Antonella Trotta, Marta Di Forti, Simona Stilo, Anna Kolliakou, Poonam Gardner-Sood, Javier-David Lopez-Morinigo, Fiona Gaughran, Anthony David, Paola Dazzan, Carmine Pariante, Valeria Mondelli, Robin Murray, Helen Fisher

**Affiliations:** Institute of Psychiatry, Psychology and Neuroscience, King’s College London

## Abstract

**Background:**

Little is known about the impact of different forms of childhood adversity (CA) on outcomes in first episode psychosis (FEP) patients beyond the first year of treatment. We investigated associations between different types of CA and 5-year outcomes in a well-characterised sample of FEP patients.

**Methods:**

A total of 237 FEP cases aged 18–65 years were followed on average for 5 years after first presentation to psychiatric services in South-London, UK. CA was assessed at service entry using the Childhood Experience of Care and Abuse Questionnaire. Using electronic clinical notes, extensive information was collected on clinical and social outcomes, service use, and self-injurious behaviours. As case analysis with missing data provides the most severely biased results we conducted multiple imputations to handle the missing data. We imputed the missing values using multiple imputations by chained equations (MICE). MICE has been shown to be a robust method for dealing with missing data across empirical and longitudinal studies.

**Results:**

72.1% of the sample reported at least one form of CA. Childhood parental separation was associated with greater likelihood of non-compliance with antipsychotic medications (OR=2.44, 95% CI=1.11–5.39), compulsory admission (OR=2.40, 95% CI=1.32–4.37), and living alone (OR=1.99, 95% CI=1.04–3.81) by the end of the follow-up. Institutional care was associated with longer total length of inpatient stay (IRR=1.34, 95% CI=1.01–1.79); parental death was associated with compulsory admissions (OR=2.87, 95% CI=1.02–8.05) during the follow-up.

**Discussion:**

Our findings suggest some specificity in the detrimental impact of CA on service use and social functioning over a 5-year period following first contact with mental health services for psychosis. Clinicians should screen patients for CA and tailor interventions accordingly to improve outcomes.

